# The nutritional and cardiovascular health benefits of rapeseed oil-fed farmed salmon in humans are not decreased compared with those of traditionally farmed salmon: a randomized controlled trial

**DOI:** 10.1007/s00394-020-02396-w

**Published:** 2020-10-05

**Authors:** Baukje de Roos, Sharon Wood, David Bremner, Shabina Bashir, Monica B. Betancor, William D. Fraser, Susan J. Duthie, Graham W. Horgan, Alan A. Sneddon

**Affiliations:** 1 Rowett Institute, University of Aberdeen, Foresterhill, Aberdeen, AB25 2ZD UK; 2 Institute of Aquaculture, Stirling University, Stirling, UK; 3 Norwich Medical School, University of East Anglia, Norwich, UK; 4 School of Pharmacy and Life Sciences, Robert Gordon University, Aberdeen, UK; 5 Biomathematics and Statistics Scotland, Aberdeen, UK

**Keywords:** Farmed fish, Fish feeds, Cardiovascular health, Omega-3 index, Vitamin D, Micronutrients

## Abstract

**Purpose:**

Farmed fish are increasingly raised on feeds containing vegetable oils, which affects their composition and possibly health properties. We investigated the effects of consuming farmed salmon, raised on different feeding regimes, on nutrient status and health outcomes in healthy subjects.

**Methods:**

Salmon were grown on feeds containing mainly fish oil (FO) or rapeseed oil (RO), resulting in an eicosapentaenoic acid (EPA) + docosahexaenoic acid (DHA) content of fillets of 2.1 or 0.9 g/100 g, respectively. In a randomized parallel controlled trial, 51 healthy subjects were allocated to consume 2 portions/week of FO salmon (*n* = 17), RO salmon (*n* = 17) or no additional salmon (Control, *n* = 17) as part of their habitual diet, for 18 weeks. We collected blood at 0, 9 and 18 weeks to measure omega-3 index (O3I) in red blood cells, plasma markers of cardiovascular risk, serum 25(OH)-vitamin D_3_ (25(OH)D_3_) and plasma trace elements.

**Results:**

After 18 weeks, O3I was similarly increased in subjects consuming 2 portions/week of FO or RO salmon compared to control (both* p* < 0.05). Serum 25(OH)D_3_ was significantly higher, whereas plasma triacylglycerols were significantly lower in subjects consuming RO salmon compared to control (both* p* < 0.05). Heart rate was significantly lower in subjects consuming FO salmon after 9 weeks, compared to control (*p* < 0.01). Salmon consumption did not affect other markers.

**Conclusion:**

Consuming two portions/week of salmon raised on rapeseed oil rather than fish oil increased the O3I and vitamin D status, and decreased plasma triacylglycerols. These outcomes endorse opportunities for developing more sustainable feeds within aquaculture food systems.

**Clinical trial registry:**

This trial was registered at clinicaltrials.gov as NCT01916434.

**Electronic supplementary material:**

The online version of this article (10.1007/s00394-020-02396-w) contains supplementary material, which is available to authorized users.

## Introduction

Worldwide, fish is a valuable dietary source of not only energy, protein and n-3 long-chain polyunsaturated fatty acids (n-3 LCPUFA), but also vitamins such as A and D, and micronutrients such as selenium, zinc and magnesium [1]. Whilst the health benefits of seafood consumption have traditionally been attributed to its content of n-3 LCPUFA [1], a recent systematic assessment of the effects of n-3 LCPUFA supplements concluded that increasing their consumption had little or no effect on mortality or cardiovascular health [2]. Nevertheless, two meta-analyses of fish intervention studies confirmed that, compared with very low fish intake (i.e., < 1 serving/month), low or moderate fish intake (i.e., 1 or 2 servings/week) significantly reduced the risk for coronary heart disease and stroke [3, 4]. Furthermore, meta-analysis of observational data indicated a moderate and inverse association between fish consumption and cerebrovascular risk, whilst this was not the case for n-3 LCPUFA status in observational studies, or supplement intake in primary and secondary prevention trials [5]. This suggests that the beneficial effect of fish intake on disease risk could be mediated through an interplay of different nutrients in fish.

The total supply of fish for food consumption has increased significantly in the past five decades due to population growth, rising incomes, urbanization and a strong expansion of global production and distribution networks. Further growth in fish consumption levels will be increasingly dependent on aquaculture. Indeed, aquaculture already makes a crucial contribution to local diets and economies in many countries [6, 7]. Expanding the aquaculture sector has the potential to reduce the burden on wild fish stocks whilst meeting the dietary needs of the population for n-3 LCPUFA and other nutrients [8]. However, there is a strong need to understand the effect of pressures from sustainability on methods of production and the health-giving properties of fish. In the past decades, the aquaculture industry has improved the sustainability of fish production by lowering the input–output ratios for salmon [9]. Traditional fish oil in formulated fish diets is increasingly being replaced by oil from plant-based sources. Among such oils, rapeseed oil is the most frequently utilized fish oil alternative in European aquaculture systems, mainly due to its ready availability and favorable price [10]. However, use of a terrestrial oil such as rapeseed may lower concentrations of the main n-3 LCPUFA, eicosapentaenoic acid (EPA) and docosahexaenoic acid (DHA), as well as other important nutrients in fish, and thereby the subsequent health benefits to the consumer [11]. Consequently, the objective of this study was to: (i) assess the effects of consuming two portions per week of farmed salmon reared on different feeding regimes on the omega-3 index (primary outcome), micronutrient concentrations and cardiovascular health outcomes, (secondary outcomes) in healthy subjects and (ii) evaluate how changes in health outcomes may be related to compositional differences of the salmon.

## Subjects and methods

### Growing of salmon

Atlantic salmon (*Salmo salar L*.) were grown by Marine Harvest (now MOWI) at Ardnish fish farm in Lochailort, United Kingdom, between April 2012 and January 2013 on fish feed that was produced to differ in n-3 LCPUFA (BioMar Ltd). One group of salmon received traditional high fish meal and high fish oil feed with high concentrations of EPA and DHA (FO group: EPA + DHA ~ 15% of total feed fatty acids); whereas, the other group of salmon were fed diets where the majority of the fish oil was replaced by vegetable oils (mainly rapeseed oil) with lower concentrations of EPA and DHA (RO group: EPA + DHA ~ 6–8% of total feed fatty acids). The composition of the fish diets and feeding schedule is outlined in Supplementary Tables 1a and 1b. After 9 months, salmon length and weight were similar between both groups (Supplementary Table 2). Fish were harvested, gutted and filleted, and two portions were cut from the center part of each salmon. The average portion size was 157.1 g (SD 43.5 g, *n* = 300) for FO salmon and 153.9 g (SD 33.6 g, *n* = 300) for RO salmon. Fillets were vacuum packed, snap frozen and stored at − 80 °C for the duration of the human intervention study. Storage of salmon at − 80 °C for 12 months did not affect the fatty acid composition of the salmon (Supplementary Fig. 1).

### Compositional analysis of salmon fillets

Routine proximate analytical procedures were used to establish the macronutrient composition of the salmon. Total fat was determined by the Soxtec method (Soxtec 2050 Auto Fat Extraction System) [12]. Protein was measured as total nitrogen by the Dumas combustion method using a Vario Max CN analyzer. The nitrogen content was multiplied by 6.25 to calculate the protein concentration [13]. For fatty acid analysis, total lipids from diet subsamples were extracted using the method of Folch [14], and total lipid was then converted to its methyl esters by direct trans-esterification using 1% sulphuric acid in methanol. The proportion of individual fatty acids in the diet was determined with an Agilent 6890 gas chromatograph fitted with a 30 m DB23 capillary column (J&W Scientific, Folsom, CA, USA) as described by us previously [15]. Trace element concentrations were determined after digestion in nitric acid using the MARS 6 digestion system (CEM Corp., Matthews, NC, USA) by inductively coupled mass spectrometry (ICP-MS) analysis as previously described [16]. The accuracy of methods was assessed using certified reference materials (skimmed milk powder, LGC Group, Middlesex, UK, and whole blood, SERO AS, Billingstad, Norway). The composition of the salmon fillets is indicated in Table 1.

**Table 1:** Levels of nutrients in salmon fillets used for the intervention

	FO salmon	RO salmon	*p *value
	(g/100 g wet weight fillet)		
Protein	19.4 ± 0.8	19.1 ± 0.5	0.39
Total *N*	3.1 ± 0.1	3.1 ± 0.1	0.39
Carbohydrates	0.2 ± 0.0	0.1 ± 0.0	0.02
Fat, total	10.8 ± 2.1	10.8 ± 1.5	0.98
Saturated fat	3.0 ± 0.6	2.3 ± 0.3	0.04
Monounsaturated fat	3.7 ± 0.7	4.7 ± 0.6	0.06
Polyunsaturated fat	4.1 ± 0.8	3.8 ± 0.5	0.56
n-6 PUFA	1.5 ± 0.1	2.1 ± 0.1	0.00
n-3 PUFA	2.6 ± 0.1	1.7 ± 0.1	0.00
EPA + DHA (% of total fat)	2.1 ± 0.4 (19.5%)	0.9 ± 0.1 (8.8%)	0.00
Moisture	63.4 ± 2.0	65.3 ± 3.1	0.30
Ash	2.2 ± 0.2	2.0 ± 0.2	0.19
Vitamin D_3_ (µg/100 g)	1.8 ± 0.1	1.4 ± 0.1	0.00
Selenium (µg/100 g)	17.8 ± 2.2	19.3 ± 4.5	0.30
Zinc (µg/100 g)	578.3 ± 108.9	622.4 ± 23.5	0.16
Magnesium (mg/100 g)	28.6 ± 3.2	26.3 ± 1.9	0.60

Values are means ± SEMs (*n* = 5 fillets)

### Human intervention study

#### Ethics

The study was carried out in accordance with the ethical principles of the Declaration of Helsinki and Good Clinical Practice. The study was approved by the Ethics Committee of the Rowett Institute, University of Aberdeen.

#### Study subjects and study design

This was a randomized parallel intervention study in healthy and free-living male and female subjects. All subjects provided informed consent and completed a health questionnaire prior to starting the study. Eligibility was established through a health questionnaire and a screening blood sample. Inclusion criteria were men and women aged 35–75 years; BMI ranging from 25–35 kg/m^2^; normal results on full blood count (haematocrit above 40% for males and above 35% for females; hemoglobin above 13 g/dL for males and above 11.5 g/dL for females); blood pressure below 160/90 mmHg; fasting plasma glucose < 7 mmol/L; normal results of fasting plasma lipids (total cholesterol < 8 mmol/L, total/HDL cholesterol < 6 mmol/L). Exclusion criteria were regularly taking aspirin or aspirin-containing drugs, or other anti-inflammatory drugs; taking drugs or herbal medicines known to alter the haemostatic system in general; taking any medicine known to affect lipid metabolism; taking dietary supplements/multivitamin tablets; diagnosis of diabetes, hypertension, renal, hepatic, hematological disease or coronary heart disease; having given a pint of blood for transfusion purposes within the last month; unsuitable veins for blood sampling; inability to understand the participant information sheet; inability to speak, read and understand the English language.

#### Study protocol

Eligible subjects were randomized to 1 of the 3 possible parallel interventions, matched for gender, age and BMI, by an independent statistician, based on the employment of covariate balancing/minimization. Subjects were then asked to either consume (i) 2 portions of FO salmon per week on top of habitual fish consumption, (ii) 2 portions of RO salmon per week on top of habitual fish consumption, or (iii) continue with habitual fish consumption (Control), for 18 weeks. Either FO or RO salmon portions were provided to the subjects in each group at the start of the study and at their 9-week study visit. The primary outcome in this study was the O3I. A daily intake of ~ 0.5 g n-3 LCPUFA was expected to increase the O3I from ~ 4% to ~ 6% after 18 weeks, and a daily intake of ~ 1 g n-3 LCPUFA would be expected to produce twice this increase [17, 18]. Five previous human intervention studies with n-3 LCPUFA showed an average SD index% between 0.78 and 0.92%, with a mean SD of change of 0.85% (data supplied by W. Harris). To detect a group difference of 1% for this SD, on the basis of a 5% significance level and a power of 90% for detecting a main effect on the omega-3 index, a sample size of 17 per each intervention and control group would be required.

Subjects attended the Human Nutrition Unit at the Rowett Institute in Aberdeen, United Kingdom, after an overnight fast of ≥ 10 h, just before the start of the study, and after 9 and 18 weeks of intervention, where their blood pressure and heart rate were measured, and a blood sample was taken. Prior to the visits at the start and during the 18th week of intervention, 4-day estimated food diaries were recorded by each participant. Blood samples (70 mL) were obtained using siliconized 21-gauge butterfly needles into vacutainers containing either EDTA as anticoagulant or vacutainer serum tubes (Becton Dickinson, Oxford, UK). Whole blood samples were used for blood cell count using a Sysmex KX21 (Sysmex Ltd, UK). 100 µL of whole blood was frozen at − 70 °C for the analysis of fatty acid composition in red blood cells (RBC). Plasma was obtained by centrifuging whole blood at 2000*g* for 15 min and stored at − 70 °C for the analysis of markers of lipid metabolism, glucose, insulin, inflammation and elements. Serum samples were collected after whole blood was left to clot for 1 h and then centrifuged for 2000*g* for 15 min. The serum was stored at − 70 °C for the analysis of 25(OH)D_3_.

Compliance was calculated at the end of the study from participant’s diaries and fish logs with the following formula: compliance (%) = number of salmon portions eaten over 18 weeks/36 (number that should have been eaten over 18 weeks). The 4-day food diaries were analyzed with the NetWISP analysis program (version 3.0) (Tinuviel Software, Llanfechell, Anglesey, UK) using the UK Nutrient Databank [19].

### Biochemical analyses

#### Fatty acid composition in red blood cells

Fatty acid composition in red blood cells (RBC) was performed as described by Harris et al. [18], using an Agilent 6890 gas chromatograph as described above [15]. The O3I was subsequently calculated as the absolute amount of EPA + DHA as percentage of total fatty acids [17].

#### Analysis of cardiovascular risk markers

Glucose, total cholesterol, HDL cholesterol, triacylglycerols and non-esterified fatty acids (NEFAs) were measured in plasma on an automated clinical analyzer (KONELAB30, Thermo Fisher Scientific, UK) using kits obtained from Microgenics GmbH, UK. LDL cholesterol was calculated with the Friedewald formula [20]. Clottable fibrinogen was measured in plasma in duplicate according to the method [21] in a semi‐automated coagulometer (Schnitger and Gross, Burkard Scientific (Sales) Ltd., UK). Soluble intercellular adhesion molecule‐1 (sICAM‐1), soluble P‐selectin (sP‐selectin) and high-sensitivity C-reactive protein (hs-CRP) were analyzed in plasma with Platinum ELISA kits (Bender MedSystems GmbH, Austria); insulin was analyzed in plasma with a Mercodia ELISA kit (Mercodia, Uppsala, Sweden). All samples were measured in duplicate.

#### Vitamin D analysis

The vitamin D metabolite 25(OH)D_3_ was analyzed in serum as described [22]. This LC–MS/MS method has previously been validated against other commercially available assays and is regarded as the most valid and reliable technique for the assessment of vitamin D metabolites including 25(OH)D_3_ [23]. All samples were measured once, with a subset of 9 samples measured in duplicate.

#### Trace element analysis

Plasma concentrations of selenium, zinc and magnesium were analyzed by diluting plasma into concentrated nitric acid (65% (v/v) and then incubating at 165 °C for 10 min followed by analysis using ICP-MS as previously described [16]. The accuracy of the method was assessed using certified reference materials: whole blood (Seronorm Whole Blood L-3) and serum (Seronorm Serum L-1) (SERO AS, Billingstad, Norway). The median recovery values of the relevant elements were within the certified ranges indicated by the supplier. All samples were measured in duplicate.

### Statistical analysis

Statistical analysis was carried out using R 3.4.0 (R Foundation for Statistical Computing, Vienna). Baseline data were analyzed by Analysis of Variance with terms for participant age, gender, BMI and treatment group. Outcome data were analyzed by Analysis of Variance with terms for participant age, gender, BMI, baseline value and treatment group. The treatment term was also split into contrasts between the control and intervention diet. We also examined analyses including interactions between treatment group and participant, age and gender, but these were not significant. Models with a time of year effect were also examined, and although these effects were significant for some outcomes, they did not affect the conclusions about treatment effects. Differences between treatment groups were tested by post hoc tests with Tukey adjustment for multiple comparisons. Results at 9 and 18 weeks are presented as separate analysis, as a repeated measures analysis did not add any additional information.

## Results

### Salmon composition

FO salmon contained 10.8 g/100 g fat of which 10.5% was EPA and 9% DHA (Table 1). Thus, two 157.1 g portions/week of FO salmon provided ~ 6.6 g of EPA + DHA per week (or ~ 0.9 g/day). RO salmon also contained 10.8 g/100 g fat of which 4.2% was EPA and 4.6% was DHA (Table 1). Thus, two 153.9 g portions of RO salmon provided ~ 2.8 g of EPA + DHA per week (or ~ 0. 4 g/day). Consumption of two portions/week of either the FO or RO salmon provided ~ 4–5% of the RDA for vitamin D, ~ 15% of the RDA for selenium, and ~ 3–6% of the RDA for zinc and magnesium (Table 1). FO salmon contained significantly higher amounts of carbohydrates, saturated fat, n-3 LCPUFA, EPA + DHA and vitamin D_3_, and significantly lower amounts of n-6 PUFA, compared with RO salmon (Table 1).

### Recruitment of subjects

**Fig. 1:**
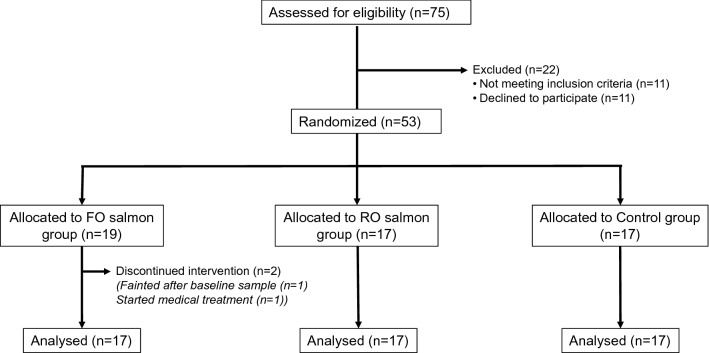
Flow chart of participant recruitment

A total of 75 subjects were recruited between November 2012 and August 2013. Of these subjects, 11 did not meet the inclusion criteria and 11 declined to participate. 53 subjects were allocated to the three intervention groups, but one participant withdrew because of fainting during blood sampling, and one participant because of an unrelated medical intervention. Therefore, 51 subjects completed the intervention with 17 subjects in each of the three intervention groups (Fig. 1). The study ended in January 2014. Baseline characteristics of the subjects did not significantly differ between these three groups (Table 2).

**Table 2:** Characteristics of subjects at baseline

	FO salmon (*n* = 17)	RO salmon (*n* = 17)	Control (*n* = 17)
Age (years)	55 ± 3	53 ± 3	54 ± 3
Sex (female/male)	10/7	10/7	11/6
BMI (kg/m^2^)	25.9 ± 1.0	25.5 ± 0.9	26.5 ± 1.0
Systolic blood pressure (mm Hg)	131.2 ± 4.2	129.2 ± 3.5	133.4 ± 3.0
Diastolic blood pressure (mm Hg)	80.7 ± 2.3	79.8 ± 1.7	78.1 ± 2.6
Heart rate (beats/min)	64.3 ± 2.3	64.3 ± 2.0	63.1 ± 3.1

Values are means ± SEMs (all such values)

### Effect of intervention on dietary intake, compliance to fish intervention and body weight

Compliance for the salmon intervention was 83% for 1 participant in the RO salmon group and 100% for the other 33 subjects. Analysis of the food diaries from the subjects from the FO salmon, RO salmon and control groups showed that there were no significant changes in habitual fish intake across the study period (data not shown). Mean intake of energy and macronutrients from the diets at baseline and after 18 weeks of intervention was not different between groups, apart from salt intake at 18 weeks, which was significantly higher in the control group than in the FO and RO salmon groups (*p* < 0.05) (Table 3). Mean body weight did not change during the intervention period in the FO salmon, RO salmon and control groups (data not shown).

**Table 3:** Mean daily intake of energy, macronutrients, salt, fibre and alcohol^a^ in subjects at baseline and after 18 weeks of intervention

	FO salmon (*n* = *15*)	RO salmon (*n* = *17*)	Control (*n* = *15*)	*p*_treatment_
Energy (kcal)				
Baseline	1897 ± 133	1775 ± 114	1898 ± 115	0.29
18 weeks	1844 ± 154	1822 ± 101	1912 ± 114	0.72
Protein (g)				
Baseline	82.0 ± 5.0	70.4 ± 4.2	79.4 ± 5.7	0.43
18 weeks	74.9 ± 5.5	71.5 ± 3.2	80.3 ± 4.6	0.18
Carbohydrates (g)				
Baseline	227.8 ± 19.3	212.4 ± 14.4	208.6 ± 16.0	0.57
18 weeks	210.9 ± 22.6	218.8 ± 13.5	204.9 ± 14.9	0.40
Total fat (g)				
Baseline	76.4 ± 5.7	69.0 ± 5.3	80.1 ± 6.5	0.19
18 weeks	76.8 ± 6.0	71.4 ± 4.8	83.1 ± 5.5	0.55
Saturated fat (g)				
Baseline	27.4 ± 2.6	26.2 ± 2.4	30.6 ± 3.2	0.29
18 weeks	27.0 ± 2.5	25.7 ± 1.9	32.0 ± 3.3	0.33
Polyunsaturated fat (g)				
Baseline	12.7 ± 1.0	10.9 ± 1.0	12.0 ± 1.4	0.49
18 weeks	12.9 ± 1.2	12.7 ± 1.3	13.2 ± 1.2	0.88
Monounsaturated fat (g)				
Baseline	22.4 ± 1.8	20.7 ± 1.6	25.1 ± 2.8	0.22
18 weeks	21.4 ± 2.1	21.3 ± 1.6	25.2 ± 1.9	0.49
Salt (g)				
Baseline	6.1 ± 0.5	6.5 ± 0.7	6.3 ± 0.6	0.89
18 weeks	5.8 ± 0.6	5.7 ± 0.4	7.2 ± 0.6	0.03
Fiber (g)				
Baseline	21.8 ± 2.0	18.7 ± 1.2	18.9 ± 1.5	0.27
18 weeks	22.0 ± 2.4	19.9 ± 1.5	20.9 ± 1.9	0.68
Alcohol (g)				
Baseline	4.6 ± 1.7	11.0 ± 2.9	11.6 ± 3.0	0.08
18 weeks	9.3 ± 3.9	10.3 ± 2.6	11.5 ± 3.0	0.84

^a^All values are means ± SEMs. Only subjects providing both food diaries (week 0 and week 18) were included in the analysis (*n* = 15, 17 and 15 in the FO salmon, RO salmon and Control group, respectively). *p*_treatment_ = *p* value for treatment effect between groups

### Effect of intervention on fish intake, the O3I and RBC fatty acid composition

**Fig. 2:**
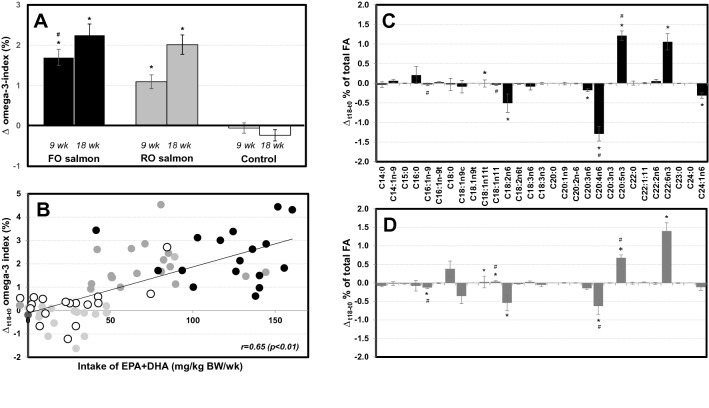
Changes in RBC fatty acid composition upon consumption of 2 portions of FO or RO salmon per week or no additional salmon (control), after 9 and 18 weeks. Data are means ± SEM. **a**: Change in O3I from baseline at 9 weeks and 18 weeks for FO salmon (black), RO salmon (gray), control (white) groups; *p < 0.01 for differences from control group; ^#^*p* < 0.05 for difference between FO and RO salmon groups. **b**: Correlation of changes in O3I with the intake of EPA + DHA per kg of body weight across the FO salmon (black), RO salmon (gray) and control (white) groups after 18 weeks of intervention. Changes in the abundance of individual fatty acids in RBC upon consumption of **c**: FO (black) or **d**: RO (gray) salmon for 18 weeks. *p < 0.05 for differences from control group; ^#^*p* < 0.05 for differences between FO and RO salmon groups

**Fig. 3:**
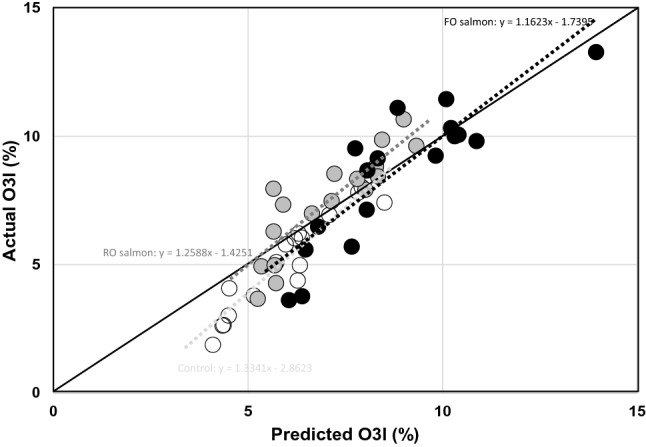
Predicted versus actual O3I (%). The predicted O3I = baseline O3I − the predicted change in O3I, with the latter being calculated with an algorithm based on studies with fish oil supplements (21): 2.60 + 0.921 × 1 – 0.842 × baseline O3I + 0.05 × (baseline O3I)^2^ + 0.0027 × [dose EPA + DHA in mg/day] – 0.000000471 × [dose EPA + DHA in mg/day]^2^. The dose of EPA + DHA/day used was 900 mg/day for the FO salmon group and 400 mg/day for the RO salmon group (see “Results”). Black circles represent the FO salmon group, gray circles the RO salmon group, and white circles the Control group. The solid line represents the line *y* = *x*, illustrating the ‘perfect prediction’. The dashed lines represent the best fit lines of the predicted compared with observed O3I values for the FO salmon group (black), the RO salmon group (dark gray) and the control group (light gray)

Mean (± SEM) weekly intake of total fish was 3.3 ± 0.1, 2.9 ± 0.1 and 1.8 ± 0.2 portions, whereas mean (± SEM) weekly intake of oily fish was 2.6 ± 0.0, 2.4 ± 0.1 and 1.0 ± 0.2, in the FO salmon, RO salmon and control groups, respectively, during the last week of the 18-week intervention. After both 9 and 18 weeks of intervention, the O3I was significantly higher in subjects consuming 2 portions/week of either FO or RO salmon, compared with the control group (both* p* < 0.05). After 18 weeks of intervention, the difference in the increase of the O3I between those consuming the FO (2.3%) and RO salmon (2.0%) was no longer significant (Fig. 2a). The changes in the O3I after 18 weeks of intervention correlated significantly with intake of n-3 LCPUFA per kg body weight (Fig. 2b). Incorporation of EPA + DHA into RBC occurred mostly at the expense of linoleic acid and arachidonic acid, and consumption of FO salmon, compared with RO salmon, led to a preferential incorporation of EPA into RBC membranes (Fig. 2c, d). Predicted O3I (%), calculated with an algorithm based on studies with fish oil supplements [24], using a dose of EPA + DHA of 900 mg/day for the FO group and 400 mg/day for the RO group (see above) showed a good alignment with observed O3I values for all three intervention groups (Fig. 3).

### Effect of intervention on cardiovascular risk markers

Plasma triacylglycerols were significantly lower in subjects consuming RO salmon, compared with the control group, after 18 weeks of intervention (*p* < 0.05) (Table 4). None of the other plasma lipids were significantly affected by the salmon intervention. Heart rate was significantly lower in subjects consuming FO salmon, compared with control, but only after 9 weeks of intervention (*p* < 0.01) (Table 4). Blood pressure and metabolic markers were not affected by the salmon intervention, although revised QUICKI, a marker of insulin sensitivity, was slightly but significantly lower in the FO group compared with the control group (*p* < 0.05) (Table 4). Unexpectedly, baseline plasma concentrations of the inflammatory markers, fibrinogen, hs-CRP and s-ICAM were significantly higher in the FO and RO groups, compared with the control group. However, inspection of relationships between baseline inflammatory markers and responses in O3I and plasma triacylglycerols did not reveal a consistent relationship between both (Supplementary Fig. 2 ).

**Table 4:** Cardiovascular risk markers^a^ at baseline and after 9 and 18 weeks of intervention

	FO salmon (*n* = 17)	RO salmon (*n* = 17)	Control (*n* = 17)	*p*_treatment_
Systolic blood pressure (mm Hg)				
Baseline	131.2 ± 4.2	129.2 ± 3.5	133.4 ± 3.0	0.896
9 weeks	130.3 ± 3.5	125.4 ± 2.8	131.5 ± 3.1	0.296
18 weeks	129.3 ± 3.5	120.8 ± 3.1	129.5 ± 3.9	0.136
Diastolic blood pressure (mm Hg)				
Baseline	80.7 ± 2.3	79.8 ± 1.7	78.1 ± 2.6	0.427
9 weeks	80.0 ± 1.9	80.2 ± 1.8	81.4 ± 2.1	0.275
18 weeks	77.9 ± 2.0	75.4 ± 2.1	77.8 ± 2.4	0.374
Heart rate (beats/min)				
Baseline	64.4 ± 2.3	64.3 ± 2.0	63.2 ± 3.1	0.592
9 weeks	58.4 ± 2.2^a^	63.9 ± 2.4^b^	65.4 ± 3.3^b^	0.002
18 weeks	61.9 ± 2.0	64.9 ± 2.8	65.0 ± 3.3	0.571
Total cholesterol (mmol/L)				
Baseline	5.42 ± 0.30	5.49 ± 0.22	5.41 ± 0.28	0.864
9 weeks	5.66 ± 0.33	5.70 ± 0.22	5.54 ± 0.27	0.889
18 weeks	5.71 ± 0.28	5.46 ± 0.17	5.60 ± 0.27	0.123
HDL cholesterol (mmol/L)				
Baseline	1.52 ± 0.08	1.74 ± 0.14	1.66 ± 0.11	0.251
9 weeks	1.68 ± 0.10	1.78 ± 0.13	1.74 ± 0.12	0.193
18 weeks	1.71 ± 0.11^a^	1.73 ± 0.15^b^	1.75 ± 0.11^a,b^	0.012
LDL cholesterol (mmol/L)				
Baseline	3.53 ± 0.26	3.33 ± 0.20	3.35 ± 0.22	0.781
9 weeks	3.53 ± 0.31	3.53 ± 0.24	3.43 ± 0.19	0.508
18 weeks	3.52 ± 0.24	3.29 ± 0.20	3.44 ± 0.21	0.393
Triglycerides (mmol/L)				
Baseline	1.35 ± 0.18	1.26 ± 0.13	1.19 ± 0.12	0.660
9 weeks	1.19 ± 0.15	1.17 ± 0.11	1.19 ± 0.15	0.533
18 weeks	1.24 ± 0.21^a,b^	1.08 ± 0.12^a^	1.31 ± 0.16^b^	0.050
Glucose (mmol/L)				
Baseline	5.53 ± 0.12	5.67 ± 0.15	5.57 ± 0.12	0.599
9 weeks	5.73 ± 0.13	5.71 ± 0.22	5.62 ± 0.11	0.711
18 weeks	5.62 ± 0.10	5.65 ± 0.18	5.66 ± 0.11	0.804
Insulin (mU/L)				
Baseline	7.71 ± 1.83	6.56 ± 0.95	6.13 ± 0.53	0.579
9 weeks	6.52 ± 0.68	6.45 ± 0.88	6.10 ± 0.64	0.717
18 weeks	6.86 ± 0.95	6.56 ± 0.80	6.37 ± 0.72	0.845
NEFA (mmol/L)				
Baseline	0.44 ± 0.04	0.44 ± 0.05	0.53 ± 0.05	0.270
9 weeks	0.44 ± 0.05	0.45 ± 0.05	0.47 ± 0.06	0.455
18 weeks	0.54 ± 0.05	0.43 ± 0.03	0.45 ± 0.04	0.055
HOMA-IR				
Baseline	1.95 ± 0.50	1.63 ± 0.21	1.53 ± 0.14	0.593
9 weeks	1.66 ± 0.17	1.65 ± 0.22	1.53 ± 0.17	0.638
18 weeks	1.66 ± 0.26	1.63 ± 0.19	1.62 ± 0.19	0.725
revQUICKI				
Baseline	0.427 ± 0.016	0.421 ± 0.009	0.414 ± 0.010	0.684
9 weeks	0.430 ± 0.014	0.424 ± 0.011	0.437 ± 0.019	0.226
18 weeks	0.404 ± 0.010^a^	0.421 ± 0.007^a,b^	0.424 ± 0.011^b^	0.041
Fibrinogen (g/L)				
Baseline	2.86 ± 0.07	2.62 ± 0.05	2.60 ± 0.10	0.006
9 weeks	2.74 ± 0.09	2.72 ± 0.08	2.66 ± 0.11	0.267
18 weeks	2.73 ± 0.10	2.69 ± 0.08	2.58 ± 0.09	0.063
hs-CRP (mg/L)				
Baseline	1.57 ± 0.69	1.68 ± 0.62	0.89 ± 0.27	0.036
9 weeks	2.89 ± 1.80	1.31 ± 0.63	1.36 ± 0.53	0.610
18 weeks	1.63 ± 0.87	1.31 ± 0.44	0.66 ± 0.26	0.596
s-ICAM (ng/mL)				
Baseline	334.5 ± 16.0	311.2 ± 16.5	280.4 ± 7.6	0.024
9 weeks	325.3 ± 18.2	318.0 ± 14.0	279.5 ± 6.4	0.274
18 weeks	332.9 ± 20.6	308.3 ± 15.5	275.1 ± 7.9	0.760
p-Selectin (ng/mL)				
Baseline	25.67 ± 2.37	25.77 ± 1.58	20.87 ± 1.17	0.100
9 weeks	24.53 ± 2.08	25.68 ± 1.57	20.95 ± 1.34	0.588
18 weeks	26.03 ± 2.21	25.03 ± 1.67	21.15 ± 1.20	0.741

^a^All values are mean ± SEM. *p*_treatment_ = *p* value for treatment effect between groups. Values with different superscript letters indicate that end values differ significantly (Tukey adjusted post hoc comparisons)

### Effect of intervention on serum 25(OH)D_3_, and plasma concentrations of trace elements

Serum 25(OH)D_3_ was significantly higher in subjects consuming RO salmon compared with the control group after 18 weeks of intervention (*p* < 0.05) (Table 5). Changes in serum 25(OH)D_3_ were also significantly correlated with changes in the O3I across all subjects (*r* = 0.33,* p* = 0.02). Plasma concentrations of selenium, zinc and magnesium were not affected after 18 weeks of intervention (Table 5).

**Table 5:** Concentration of serum 25(OH)D_3_ and plasma micronutrients^a^ at baseline and after 9 and 18 weeks of intervention

	FO salmon (*n* = 17)	RO salmon (*n* = 17)	Control (*n* = 17)	*p*_treatment_
Serum 25(OH)D_3_ (nmol/L)				
Baseline	65.91 ± 7.49	49.96 ± 7.00	48.74 ± 5.27	0.155
9 weeks	63.13 ± 5.09	66.11 ± 10.24	50.29 ± 4.69	0.190
18 weeks	78.05 ± 7.21^a,b^	86.14 ± 10.55^a^	57.76 ± 4.58^b^	0.043
Plasma selenium (µg/L)				
Baseline	78.88 ± 2.77	73.10 ± 2.45	77.39 ± 2.91	0.304
9 weeks	79.37 ± 3.67	76.84 ± 2.00	79.10 ± 3.38	0.713
18 weeks	81.45 ± 3.07	76.65 ± 2.70	79.15 ± 3.44	0.921
Plasma zinc (µg/L)				
Baseline	990.1 ± 86.8	1170.6 ± 65.8	984.5 ± 52.4	0.058
9 weeks	965.9 ± 84.9	1191.7 ± 88.1	985.4 ± 81.8	0.567
18 weeks	1083.7 ± 138.5	1360.8 ± 140.1	1024.6 ± 78.2	0.398
Plasma magnesium (mg/L)				
Baseline	18.76 ± 0.46	18.85 ± 0.37	18.90 ± 0.49	0.983
9 weeks	18.39 ± 0.51	18.79 ± 0.39	18.28 ± 0.40	0.475
18 weeks	19.05 ± 0.51	18.79 ± 0.31	18.64 ± 0.39	0.615

^a^All values are mean ± SEM. p_treatment_ = *p* value for treatment effect between groups. Values with different superscript letters indicate that end values differ significantly (Tukey adjusted post hoc comparisons)

## Discussion

In this study, consumption of 2 portions of farmed salmon per week, raised on fish oil or rapeseed oil-based feeds, increased the O3I to a similar extent after 18 weeks. Consumption of salmon raised on fish oil-based feed significantly decreased heart rate, but only after 9 weeks, and consumption of salmon raised on rapeseed oil-based feed significantly increased serum levels of 25(OH)D_3_ and decreased plasma triacylglycerols after 18 weeks of intervention. Thus, the nutritional and cardiovascular health benefits of rapeseed oil-fed farmed salmon in humans were not less significant than those of traditionally farmed salmon, despite containing only half the amounts of EPA and DHA.

Two other studies assessed the cardiovascular health benefits of consuming differently fed farmed fish. Consumption of Atlantic salmon grown on a 100% fish oil, but not on rapeseed oil, significantly increased serum n-3 PUFA concentrations and decreased serum triacylglycerols in coronary heart disease patients after 6 weeks [25]. Consumption of farmed trout raised on a marine but not plant-based feed significantly increased the O3I in healthy men after 8 weeks [26]. However, the duration of both studies may have been too short to reveal the cardiovascular health benefits of consuming fish raised on plant-based feeds. Whilst it takes days-to-weeks for maximal fatty acid incorporation into transport pools including serum/plasma, this can take weeks-to-months for functional pools like RBC [27], and this likely affects the time scale on which beneficial changes to cardiovascular health outcomes occur. In our study, the effects on the O3I and plasma triacylglycerols were more pronounced after 18 compared with 9 weeks of intervention, especially in the RO salmon group. After 18 weeks, the difference in O3I between the FO and RO salmon groups was no longer significant, although the RBC pool may not be as useful as plasma phosphatidyl choline and platelet pools for discriminating between different doses of n-3 LCPUFA [27].

The efficacy of farmed salmon or omega-3 supplements to increase the O3I appear similar (Fig. 3), indicating that changes in the O3I can be explained by intake of EPA and DHA, and that the bioavailability of n-3 LCPUFA from fish and supplements are comparable. The health implications of eating two oily fish per week are potentially significant, as previous studies have demonstrated that an O3I of ≥ 8% is associated with a lower risk of death from cardiovascular disease [28] and death from all causes [29]. Consumption of two portions/week of RO salmon also beneficially lowered plasma triacylglycerols. Other studies have found similar effects on serum/plasma triacylglycerols after shorter intervention periods, indicating that such effects are established within a couple of weeks, but mostly with relatively high intakes of fish (> 500 g/week). A 4-week intervention with 750 g/week of fatty fish in healthy subjects decreased serum triacylgycerols compared with lean meat [30]. Similarly, a 4-week lean-seafood intervention, supplied as 60% of total dietary protein, decreased fasting and postprandial serum triacylglycerols compared with a non-seafood intervention as part of a controlled diet in healthy subjects, despite the fact that both diets contained similar amounts of n-3 LCPUFA. Lowered serum triacylglycerol concentrations were associated with decreased fasting triacylglycerols in chylomicrons and VLDL, reduced fasting VLDL particle size and a reduced postprandial concentration of medium-sized VLDL particles [31]. In our study, we also found that consumption of FO salmon significantly decreased heart rate, but only after 9 weeks of intervention. Two meta-analyses of RCTs found that consumption of fish oil supplements reduced heart rate [32, 33], an effect that appeared to be predominantly attributable to DHA [32]. Interestingly, fish oil reduced heart rate particularly in those with higher baseline heart rate and those on longer treatment duration [33]; whereas, our findings provide evidence for a temporary decrease in heart rate. This may indicate that consumption of fish oil supplements rather than fish is more effective in modulating heart rate, and therefore risk of cardiovascular mortality, in the long term.

Fish, especially oily fish such as salmon, is also a major dietary source of vitamin D [34] and in our study, serum 25(OH)D_3_ concentrations were significantly correlated with the O3I. A recent meta-analysis found that consumption of at least two fish meals over 4 weeks was associated with a significant increase in 25(OH)D concentrations of ~ 4.4 nmol/L, albeit that consumption of fatty fish, longer study durations and lower baseline 25(OH)D concentrations were associated with larger increases in 25(OH)D concentrations [35]. However, increased consumption of (mostly farmed) fish did not affect plasma 25(OH)D concentrations despite increased vitamin D intake in another study [36]. In our study, which was not controlled for time of year and, thus, exposure to sunlight, subjects who consumed the RO salmon significantly improved their vitamin D status compared with subjects in the control group. However, FO and RO salmon provided approximately 0.8 and 0.6 µg/day of vitamin D_3_, respectively, which is well below the recommended 10 µg/day in the UK, or 10–20 µg/day in the USA. Therefore, increasing consumption of farmed salmon alone may not be sufficient to optimize vitamin D status [35]. Furthermore, vitamin D_3_ levels in aquaculture feeds have fallen in the past decades [37], as fish feeds with terrestrial components are generally devoid of this vitamin, with levels in farmed salmon being 25% lower than in wild salmon [34]. Therefore, this study highlights the importance of optimizing levels of vitamin D and other micronutrients in fish feeds to enhance the nutritional value of farmed fish to consumers in the future.

A growing number of studies have highlighted the contribution of fish consumption to adequate intake of micronutrients on a global scale [38, 39], although the actual contribution of fish to individual micronutrient status is debated. Despite increased total fish consumption in Bangladesh in the past two decades, mostly through increased consumption of farmed fish, iron and calcium intake from fish have decreased, whilst no significant changes in intakes of zinc, vitamin A and vitamin B_12_ have occurred, possibly reflecting lower overall nutritional quality of farmed fish [40]. Indeed, selenium levels in farmed salmon raised on increased levels of terrestrial-based diets have been shown to decrease by 50% [41]. In our study, we found no effect of salmon consumption on plasma concentrations of selenium, zinc or magnesium, which is perhaps expected considering the relatively low contribution of the salmon intervention to the RDA for these micronutrients.

This study had several strengths, including its interdisciplinary farm-to-fork design to link sector-representative fish feed formulations to key human health outcomes. Another strength is the intervention amount, which is in line with dietary guidelines, and the relatively long study duration, allowing the assessment of both short and longer-term effects. Study limitations include a relatively small study population of healthy Caucasian subjects, which limits the extrapolation of findings to other relevant groups such as those at risk for cardiovascular disease. Another limitation is that power analysis was only performed for the primary outcome (omega-3 index) and that significant changes in serum 25(OH)D_3_ and plasma triacylglycerol concentrations, and heart rate, could have been due to chance. However, these changes are in line with those expected based on findings in previous studies.

In conclusion, this study provides robust evidence for the cardiovascular health benefits of consuming farmed salmon. Moreover, our findings show that introduction of more sustainable feeding regimes in the aquaculture sector does not necessarily compromise the health benefits of farmed salmon, as long as two portions/week are consumed.

## Electronic supplementary material

Below is the link to the electronic supplementary material.

Supplementary file1 (DOCX 384 kb)

## Abbreviations

25(OH)D_3_: 25(OH)-vitamin D_3_
CVD: Cardiovascular disease
FO salmon: Salmon fed a fish oil-rich diet
hs-CRP: High-sensitivity C-reactive protein
ICP-MS: Inductively coupled plasma mass spectrometry
n-3 LCPUFA: N-3 long-chain polyunsaturated fatty acids
NEFA: Non-esterified fatty acids
O3I: Omega-3-index
RCT: Randomized controlled trial
RO salmon: Salmon fed a rapeseed oil-rich diet
s-ICAM: Soluble ICAM
sP‐selectin: Soluble P‐selectin
