# Supplementary material for: The nutritional and cardiovascular health benefits of rapeseed oil-fed farmed salmon in humans are not decreased compared with those of traditionally farmed salmon: a randomized controlled trial
Source: Eur J Nutr. 2020 Oct 5;60(4):2063–75. doi: 10.1007/s00394-020-02396-w (PMC8137615; doi:10.1007/s00394-020-02396-w)
Supplement: Supplementary file 1 — Supplementary file1 (DOCX 384 kb) [file 394_2020_2396_MOESM1_ESM.docx]

**Supplemental Table 1a**. Fish feed composition

| Feed pellet size | **3mm** | | **4.5mm** | | **6.5mm** | | **9mm** | | **12mm** | |
| --- | --- | --- | --- | --- | --- | --- | --- | --- | --- | --- |
| Salmon group | **FO** | **RO** | **FO** | **RO** | **FO** | **RO** | **FO** | **RO** | **FO** | **RO** |
|  | (%) | | | | | | | | | |
| Fish meal | 40.8 | 40.8 | 42.5 | 42.5 | 35.7 | 35.7 | 21.0 | 21.0 | 24.5 | 24.5 |
| Soya cake | 3.8 | 3.8 | 4.5 | 4.5 | 4.5 | 4.5 | 6.0 | 6.0 | 6.0 | 6.0 |
| Wheat gluten | 2.5 | 2.5 | - | - | - | - | 7.0 | 7.0 | 8.0 | 8.0 |
| Sunflower cake | 11.7 | 11.7 | 11.5 | 11.5 | 13.0 | 13.0 | 13.0 | 13.0 | 10.4 | 10.4 |
| Corn gluten | 7.7 | 7.7 | 5.3 | 5.3 | 8.0 | 8.0 | 15.0 | 15.0 | 10.0 | 10.0 |
| Fava beans | 13.0 | 13.0 | 14.5 | 14.5 | 13.0 | 13.0 | 6.5 | 6.5 | 6.5 | 6.5 |
| SH fish oil | 18.9 | 8.9 | 20.1 | 9.1 | 24.1 | 11.1 | 8.5 | 2.5 | 8.2 | 2.1 |
| Rapeseed oil | - | 10.0 | - | 11.0 | - | 13.0 | 9.5 | 15.7 | 11.0 | 17.8 |
| Palm olein | - | - | - | - | - | - | 2.6 | 8.5 | 4.0 | 9.8 |
| Standard fish oil | - | - | - | - | - | - | 7.5 | 1.4 | 8.5 | 2.0 |
| Supplements | 2.7 | 2.7 | 2.7 | 2.7 | 2.7 | 2.7 | 4.8 | 4.8 | 4.3 | 4.3 |

*FO: fish oil-fed salmon; RO: rapeseed oil-fed salmon; SH fish oil: Southern Hemisphere fish oil.*

**Supplemental Table 1b**. Feeding schedule

| **Diet type** | **Dates fed** | **Feeding period** |
| --- | --- | --- |
| 3 mm | 04 May 2012 – 14 Jun 2012 (6 wk) | 6 wk |
| 4.5 mm | 15 Jun 2012 – 23 Aug 2012 (10 wk) | 10 wk |
| 6 mm | 24 Aug 2012 – 10 Oct 2012 (7 wk) | 7 wk |
| 9 mm | 11 Oct 2012 – 15 Oct 2012 (1 wk) | 1 wk |
| 6 mm | 16 Oct 2012 – 11 Nov 2012 (3.5 wk) | 3.5 wk |
| 12 mm | 12 Nov 2012 – 5 Dec 2012 (3.5 wk) | 3.5 wk |
| 9 mm + 12 mm | 6 Dec 2012 – 18 Dec 2012 (2 wk) | 2 wk |
| 4.5 mm + 12 mm | 19 Dec 2012 – 07 Jan 2013 (2.5 wk) | 2.5 wk |

**Supplemental Table 2**. Salmon length and weight during the fish feeding period

|  |  | FO salmon | RO salmon | p-value |
| --- | --- | --- | --- | --- |
| *Baseline (11 May 2012)* | | | | |
|  | Weight (g) (n=100) | 102.0 ± 2.2 | 95.1 ± 2.4 | 0.037 |
|  | Length (cm) (n=25) | 21.9 ± 0.3 | 20.5 ± 1.0 | 0.182 |
| *Midpoint (27 September 2012)* | | | | |
|  | Weight (g) (n=100) | 1.2 ± 0.0 | 1.2 ± 0.0 | 0.128 |
|  | Length (cm) (n=50) | 43.6 ± 0.4 | 45.2 ± 0.4 | 0.004 |
| *Final (7 January 2013)* | | | | |
|  | Weight (g) (n=25) | 2.4 ± 0.1 | 2.5 ± 0.1 | 0.457 |
|  | Length (cm) (n=25) | 56.4 ± 0.6 | 56.8 ± 0.9 | 0.723 |


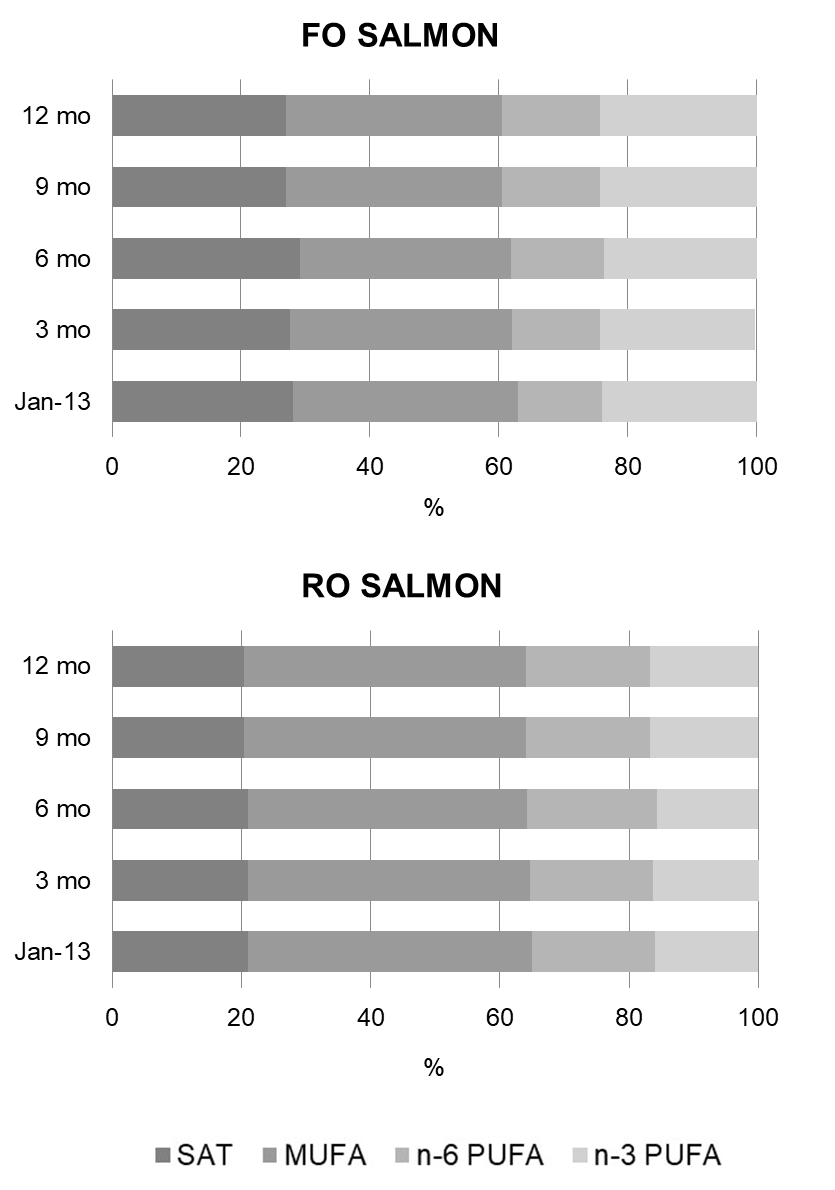


**Supplemental Figure 1**. Effects of storage on the fatty acid composition of the fish-oil (FO) and rapeseed oil (RO)-fed salmon. Figures indicate saturated fatty acids (SAT), monounsaturated fatty acids (MUFA), n-6 polyunsaturated fatty acids (n-6 PUFA) and n-3 polyunsaturated fatty acids (n-3 PUFA) as percentage of total fatty acids at harvest (January 2013) and after 3, 6, 9 and 12 months of storage at -80°C.


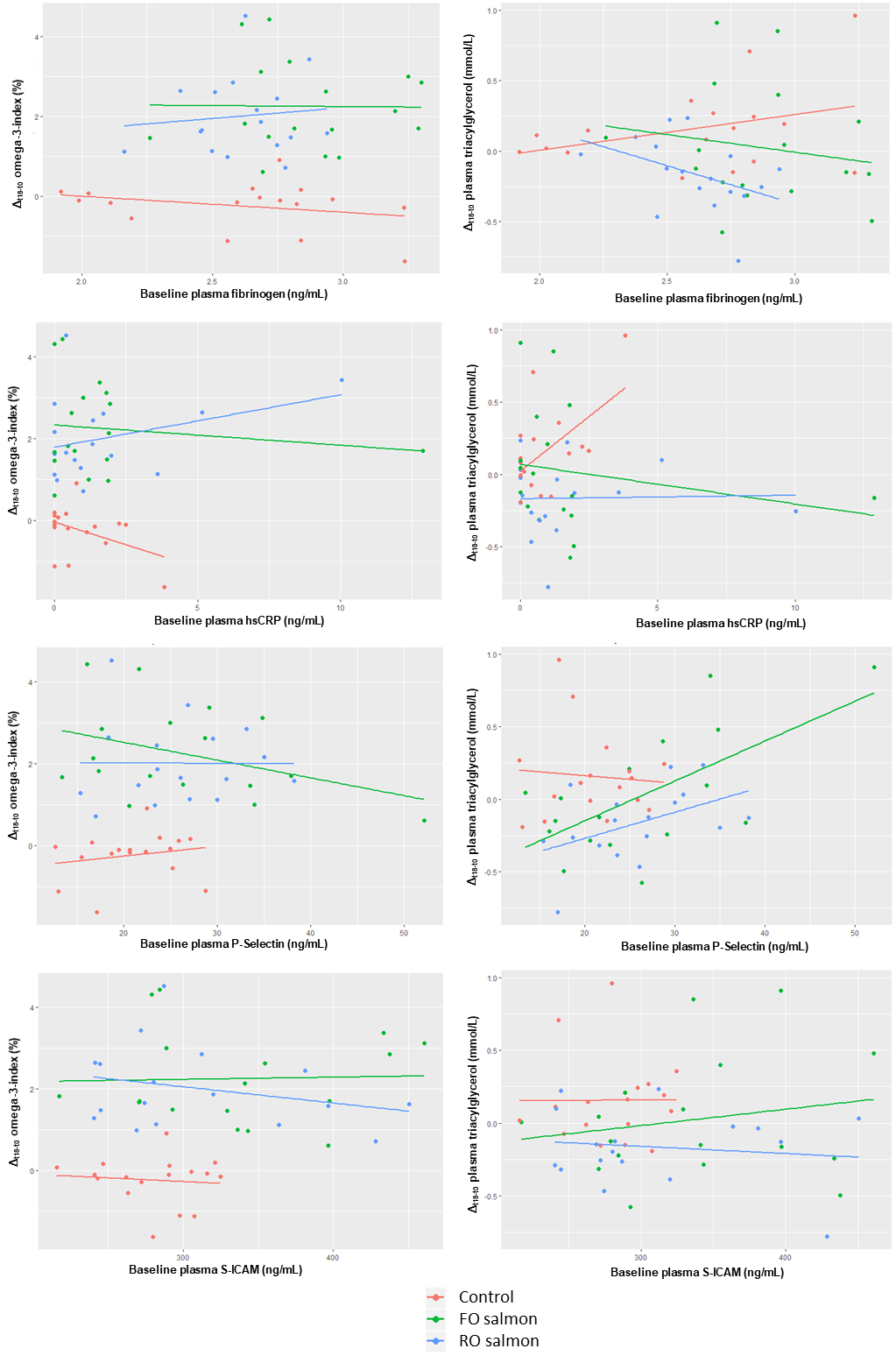


**Supplemental Figure 2**. Relationship between plasma inflammatory markers at baseline and responses in O3I and plasma triacylglycerols
